# Implementing a patient-oriented discharge summary to improve hospital-to-home transitions in older adults: lessons from a hybrid study

**DOI:** 10.3389/frhs.2025.1730127

**Published:** 2026-01-16

**Authors:** Joanie Pellet, Raquel Solano Araujo, Saganah Kathirkamu, Roger Hilfiker, Nicole Bartholdi, Cedric Mabire

**Affiliations:** 1 Institute of Higher Education and Research in Healthcare, Lausanne University Hospital and University of Lausanne, Lausanne, Switzerland; 2 Healthcare Direction, Lausanne University Hospital, Lausanne, Switzerland; 3 Patient Research Partner, Onnens, Switzerland

**Keywords:** caregivers involvement, hospital discharge, hybrid type II design, implementation science, older adults, patient-centered care, patient-oriented discharge summary, transitional care

## Abstract

**Introduction:**

Hospital discharge is a vulnerable transition for older adults who often leave with limited understanding of their health and care instructions. This study evaluated the implementation and outcomes the Patient-Oriented Discharge Summary (PODS), a one-page co-designed tool to support hospital-to-home transitions.

**Methods:**

Using a hybrid type II design, we combined a quasi-experimental pre–post study with an implementation evaluation in a Swiss acute care unit. Patients aged ≥50 years discharged home were allocated to control (*n* = 55) or intervention (PODS; *n* = 56). The primary outcome was perceived quality of care transition measured using the Care Transition Measure (CTM-15). Implementation outcomes were assessed through surveys, focus groups and interviews with healthcare professionals.

**Results:**

PODS participants reported higher CTM-15 scores than controls (74.4 vs. 62.3, *p* < 0.001). Implementation findings showed that the PODS structured discharge teaching and supported dialogue but its blank, collaboratively completed format led to variable completeness and limited usefulness at home. Persistent barriers included workload, workflow integration, and uneven interprofessional engagement.

**Conclusions:**

PODS improved perceived quality of care transition, primarily through the relational and educational processes it structures rather than the written document alone. While valuable, PODS alone appears insufficient; combining structured tools with contextual and organizational supports may enhance effectiveness.

**Clinical Trial Registration:** clinicaltrials.gov, identifier (NCT06123546).

## Introduction

Despite the importance of hospital discharge as a critical transition point, many older adults leave hospital with a limited understanding of their diagnosis, medications, follow-up, or warning signs (1). The growing complexity of chronic conditions, shorter hospital stays, and cognitive or emotional overload at discharge make it increasingly difficult for patients and caregivers to process and retain key information discussed with healthcare professionals. Studies show that 40% to 80% of patients forget or misunderstand what was discussed during their hospital stay (2, 3). This limited recall has been linked to reduced self-management capacity and higher post-discharge healthcare utilization, including emergency visits and readmissions (4–6).

For older adults, understanding discharge instructions can be affected by age-related cognitive changes, fatigue, emotional stress, and limited health literacy (2, 31). On the healthcare professional side, time constraints, lack of structured tools, and insufficient involvement of family caregivers can make it challenging to deliver clear and useful information (32–36). Traditional discharge summaries are typically written by physicians for other healthcare providers, focusing on clinical details such as diagnoses, procedures, and medication changes. In Switzerland, discharge summaries are usually prepared by physicians using standardized templates provided by each institution. They are primarily intended for the patient's general practitioner (GP) and are typically transmitted electronically through secure communication channels. A standardized medico-social transmission document is also completed by ward nurses for home care services and transmitted electronically. Patients may request a copy of these documents, but they are often lengthy, use medical jargon, and rarely address what matters most to patients—such as who to contact if problems arise or how and when to take new medications.

Bridging these gaps requires discharge tools designed with and for patients (29), ensuring that information is communicated in a way that is understandable, relevant, and actionable once patients return home. Responding to this need, the Patient-Oriented Discharge Summary (PODS) was co-developed by patients and healthcare professionals in Canada to bridge communication gaps and promote continuity of care after hospitalization (7). The PODS is a one-page summary that supports discharge teaching and follow-up planning through clear, patient-friendly language and structured sections covering the reason for hospitalization, warning signs to monitor, key contacts, treatment plan, and upcoming appointments (7). The PODS is completed collaboratively between multiple healthcare professionals and the patient before discharge, serving as both a teaching aid during hospitalization and a reference tool once back home. Rather than serving primarily as a medical record, the PODS functions as a communication and teaching tool that supports patient engagement, comprehension, and self-management during the transition home.

Initial studies have shown that PODS improves patient satisfaction with the discharge process and readiness for discharge (24, 26, 37). From the perspective of professionals, the tool has been found to be easy to use, without significantly increasing workload, feasible to integrate into routine practice, and valuable in structuring discharge conversations (37, 38). Building on this preliminary evidence, it was relevant to investigate whether the PODS can facilitate smoother hospital-to-home transitions for older adults in French-speaking Switzerland, and to examine its implementation in routine clinical practice. Unlike previous studies that tested pre-filled or standardized versions of the PODS (26–28), we implemented a blank, collaboratively completed template to promote co-production and individualization, but it also raises questions regarding feasibility, fidelity, and post-discharge usefulness. Therefore, this study aimed to evaluate the effectiveness of a patient-oriented discharge summary (PODS) in improving older adults' perceived quality of the transition from hospital to home. Secondary outcome is problems and difficulties experienced by older adults during the first ten days after discharge. In parallel, this study assessed the implementation of this tool within an acute care setting, focusing on its implementation determinants, feasibility, acceptability, and integration into routine clinical practice.

## Materials and methods

### Study design

**Figure 1:**
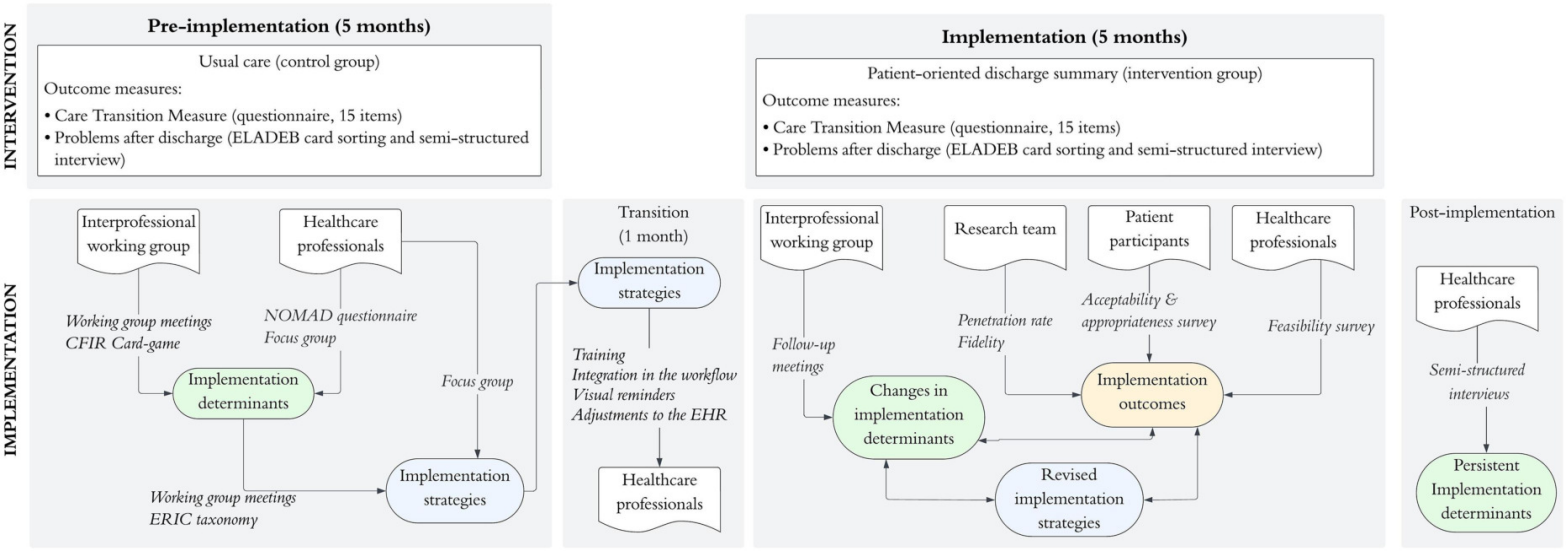
Overview of the study component.

A hybrid type II design combined a quasi-experimental pre-post study with a multi-methods implementation evaluation. The article is structured in accordance with the Standards for Reporting Implementation Studies (StaRI) Statement (8). Figure 1 provides an overview of the study method, including both the intervention components and the implementation process.

### Study setting and participants

The study was conducted in a 40-bed medical unit of a regional hospital in French-speaking Switzerland. The control phase was conducted from June to October 2024, followed by the intervention phase from November 2024 to March 2025. The target population for the intervention consisted of older inpatients, meeting the following inclusion criteria: aged ≥50 years, discharged to home planned at the beginning of the hospitalization, hospitalized for more than 48 h, able to read and speak French, and capable of providing written informed consent. We used a ≥50-year age criterion because multimorbidity is increasingly common from mid-life onward in Switzerland, and a higher age cutoff would have substantially reduced recruitment, as many adults over 65 are typically discharged to rehabilitation rather than directly home. A minimum 48-hour stay was required to ensure sufficient exposure to ward care and, in the intervention arm, time to initiate the PODS. Patients were excluded if, according to the healthcare team, they were unable to follow the study procedures due to cognitive impairment or language barriers.

The implementation component of the study targeted the unit's healthcare professionals who were expected to help patients complete the PODS, which included nurses, physicians, liaison nurses, dietitian, occupational therapists and physiotherapists.

### Recruitment

A total sample size of 130 participants (65 in the control group and 65 in the intervention group) was determined to detect a mean difference of 10 points on the primary outcome measure (assumed SD = 18, β = 0.80, α = 0.05), accounting for a 20% attrition rate. For both groups, newly admitted patients were routinely screened for eligibility by the on-site project lead, nurse managers, or clinical nurse specialists, who informed the bedside nurses accordingly and the research collaborator for information and consent.

A working group composed of professionals from the unit was established to support the implementation process and served as a key source of data. The group was formed by the on-site project lead—appointed by the nursing directorate in collaboration with the unit nurse manager—together with the nursing directorate. Professionals from each discipline were identified and approached by the project lead to join the group: two physicians, three clinical nurse specialists, two staff nurses, one healthcare assistant, one physiotherapist, one dietitian, one liaison nurse, and the on-site project lead herself, who was an assistant nurse manager. Over the course of the implementation, additional members were invited as needed to ensure continued representation across all disciplines, and all team members were invited to complete the questionnaire for assessing factors influencing the implementation of complex interventions and study surveys on the feasibility and fidelity of the intervention. For qualitative data collection related to implementation (focus groups, interviews), participants were identified by the nurse manager and selected based on their availability on the chosen dates, while ensuring maximum possible representation of the different professional disciplines.

### Intervention

The intervention is reported in accordance with the Template for Intervention Description and Replication (TIDieR) checklist (39). It took place in an acute care unit of a regional hospital in Switzerland and was designed to structure discharge teaching, strengthen patient understanding, and involve caregivers. The Patient-Oriented Discharge Summary (PODS) constituted the core component of the intervention, complemented by the systematic use of teach-back and the involvement of caregivers. To ensure consistent use of the PODS, the healthcare team was provided with various resources, including posters summarizing the PODS content and standardized workflow, a training video developed by the working group, examples of completed PODS, refresher discussions during team meetings and visual reminders in nurses office and health electronic record. To support consistent implementation, the nurse manager reminded the team each morning about which patients had an active PODS.

The PODS sections were based on the official French translation provided in the PODS Toolkit (https://pods-toolkit.uhnopenlab.ca). We used this version as a starting point and conducted a cultural adaptation for our local context, further refining the wording and the layout in collaboration with the senior patient partner and the unit's healthcare team (Supplementary Material S1). The senior patient partner was actively involved in the research project as a member of the research team. She contributes on an *ad hoc* basis, particularly when a patient perspective is needed to inform specific components of the intervention or study procedures. The PODS contained six sections of information to be completed: (1) reason for hospitalization (2) health problems; (3) warning signs and symptoms to watch for, (4) changes in daily activities and health behaviors, (5) upcoming follow-up appointments, and (6) contact information. The bedside nurse was responsible for providing the PODS to the participants and explaining the purpose of the document. The PODS was kept in the participants' room. The intervention was delivered throughout the patient's hospital stay rather than in a fixed number of sessions. The intervention was delivered face-to-face at the bedside by multiple healthcare professionals, each responsible for specific sections of the PODS based on their clinical role. The bedside nurse responsible for admission introduced the PODS and explained its purpose. Throughout the hospital stay, nurses progressively completed the PODS during routine care encounters and based on daily medical rounds, particularly for the sections related to health problems and symptoms. Physiotherapists added information related to mobility, physical activity, and functional recommendations when physiotherapy sessions occurred. Dietitians contributed to the section on lifestyle and dietary behaviours when clinically indicated. Near the end of the stay, liaison nurses completed or verified the contact information and community follow-up arrangements. Physicians contributed content for warning signs and symptoms requiring medical attention, and nurses added details regarding follow-up appointments. On the day of discharge, the bedside nurse was responsible for reviewing and verifying the information recorded in the PODS.

The frequency and duration of these interactions varied depending on patients' needs, resulting in a variable “dose” across participants. The intervention was intentionally designed to be highly personalized, with all PODS sections completed using patient-specific information. Using a blank template was intentionally employed to encourage active patient participation. By progressively filling out the PODS during the hospitalization, it functioned not only as an information record but also as a pedagogical tool: patients engaged in recalling, reformulating, and co-producing key information, which supported memory retention and ownership of their discharge plan. The teach-back technique was systematically integrated into this process: when reviewing PODS content, healthcare providers were also reminded to use the teach-back principles. This allowed misunderstandings to be identified and corrected immediately. Whenever possible, caregivers were identified early during admission and invited to participate in the process. Their role in supporting both patient comprehension and post-discharge care was emphasized during staff training.

No substantial modifications were made to the intervention during the study period.

### Implementation process

The implementation followed the EPIS framework (Exploration, Preparation, Implementation, Sustainment) (9). The preparation phase took place from February 2024 to October 2024, while the implementation phase was conducted from November 2024 to March 2025. An on-site project lead and an interdisciplinary working group composed of staff members were designated to guide the implementation process. Together with the research team, this working group also contributed to identifying and monitoring implementation determinants and selecting the implementation strategies that would address the determinants.

The preparation phase involved maintaining the usual discharge practices while recruiting and collecting data from participants in the control group. During ths phase, a contextual analysis of the unit was conducted using the Consolidated Framework for Implementation Research 2 (CFIR2) with the interdisciplinary working group in a collective format to identify potential barriers and facilitators (10). To facilitate the identification of barriers and facilitators, the study investigator used the CFIR Card Game, a structured tool that presents CFIR constructs as individual cards (11). Healthcare professionals from the working group discussed how these constructs applied to their setting, encouraging collaborative reflection and dialogue between the clinical and research teams. To confirm or challenge the implementation determinants identified through the card game session, a first semi-structured focus group was conducted in the unit by the investigator and the research collaborator with other staff members, including nurses, liaison nurses, physiotherapists, physicians (Supplementary Material S2). All members of the unit team were also invited to complete the Normalization Measure Development (NoMAD) questionnaire, a validated tool for assessing factors influencing the implementation of complex interventions from the perspective of healthcare professionals (12, 13).

Based on the findings from the contextual analysis, the implementation strategies guiding the project were identified and then mapped to the Expert Recommendations for Implementing Change (ERIC) taxonomy (14). Regular workshops held with the interprofessional team as part of the project management approach offered a structured space to collaboratively identify implementation strategies. Some were primarily initiated and led by the research team, while others were driven by hospital leadership and unit staff. To validate and refine the strategies developed by the working group, the investigator and the research collaborator held a second focus group with unit staff members to confirm alignment with their needs and incorporate complementary perspectives (Supplementary Material S2). Several strategies were designed and deployed prior to implementation and as the implementation unfolded, additional strategies were developed or adjusted in response to emerging barriers and facilitators.

At the end of the preparation phase, the unit team received formal training in using the PODS. During the implementation phase, all patients aged 50 years and older who were admitted to the unit, planned for discharge home, and whom the on-site project lead and nurse manager considered appropriate, received the PODS within 24 h of admission. Implementation determinants and outcomes were continuously monitored and shared with the working group. Monthly follow-up meetings provided an ongoing feedback loop and enabled the refinement of the implementation strategies as needed.

The implementation phase lasted 5 months. At the end of this phase, four interviews were conducted by the investigator and the research collaborator to identify persistent obstacles to PODS implementation. These interviews were conducted with a physiotherapist, two nurses and a nurse clinician (Supplementary Material S2).

### Measures

#### Intervention outcomes

Sociodemographic and clinical data were collected from electronic health records (EHR) and patient self-reports, including age, sex, number of medications (Anatomical Therapeutic Chemical Classification System—ATC), activities of daily living (ADL) (15), reason for hospitalization, length of stay, living situation (living alone or not), educational level, and number of hospitalizations in the past 12 months.

The primary outcome was the quality of care transition, measured using the Care Transition Measure Tool (CTM-15) at 5–10 days post-discharge (16). The CTM is a 15-item self-report questionnaire that evaluates the quality of the posthospital care transition experience from the patient's perspective in four factors: critical understanding, preferences important to them, management preparation, and care plan (16). Responses for each item ranged from 1 = “strongly disagree” to 4 = “strongly agree”. The mean total score was calculated by adding the value of each responded item and dividing this score by the number of answered items. The score was converted on a 0–100 scale using the formula: [(mean score—1)/3]*100. Cronbach's alpha for the CTM-15 is 0.93 (23). Construct validity showed that CTM scores had a small negative correlation with age (*r* = −0.16, *p* = 0.03) and length of stay (*r* = 0.14, *p* = 0.05) (16). The CTM-15 has been translated into French according to the principles of good practice for translation and cultural adaptation (17).

Participants were invited to complete the CTM-15 questionnaire 5–10 days after returning home. Prior to discharge, the research collaborator discussed with each participant to identify their preferred method for completing the questionnaire, with the goal of maximizing response rates and minimizing the dropouts. Participants could choose to return the completed paper questionnaire by mail (a pre-stamped envelope was provided), by email, complete it over the phone with research team support, or schedule an in-person appointment to fill it out. All responses were entered into the REDCap software for data management and analysis (18).

Problems and unmet needs were explored through face-to-face interviews conducted by the research collaborator with a subgroup of participants five to ten days after discharge, in addition to the completion of the CTM-15. The French tool Lausanne Self-Assessment Scales of Difficulties and Needs (*Échelles Lausannoises d' Auto-Évaluation des Difficultés et des Besoins*, ELADEB) was used to help participants report perceived difficulties using a card sorting method (19). Participants were presented with 21 illustrated cards, each representing a specific life domain, and asked to sort them into two categories: “Problem” or “No problem”, based on their experience after hospital discharge. This sorting exercise was followed by a semi-structured discussion to explore the reasons behind their choices and gain insight into the nature of the challenges encountered during the transition back to independent living or in adapting to new caregiving arrangements. The interviews were audio-recorded, transcribed verbatim, and a thematic analysis was conducted, which will be reported elsewhere. The results of the card-sorting activity were documented and entered into the REDCap software platform (18).

#### Implementation evaluation

##### Implementation determinants

The prospective contextual analysis used multiple data sources: the CFIR card game with the working group, two focus groups with the unit team, regular working group meetings, and the NoMAD questionnaire. The French version of the NoMAD questionnaire (12, 13) was used to assess the factors influencing the implementation of the PODS intervention from the perspective of healthcare professionals. This 20-item instrument, grounded in Normalization Process Theory, measures four constructs: coherence, cognitive participation, collective action, and reflexive monitoring. The original validation study demonstrated satisfactory construct validity, acceptable to good internal consistency across subscales (Cronbach's α = 0.71–0.81), and high internal consistency for the total scale (α = 0.89), indicating reliable measurement across contexts (12, 13). The questionnaire was administered as an online survey to all members of the unit's healthcare team after they were informed about the project.

##### Implementation outcomes

The implementation outcomes were selected based on the taxonomy proposed by Proctor et al. (20), and we retained those that were most relevant and feasible to assess given the timing and stage of the implementation process (21).

Feasibility was assessed through online surveys administered to all healthcare professionals at three time points: T1 (start of implementation), T2 (after three months of using the discharge summary), and T3 (after five months). Team members were asked whether the discharge summary increased their workload, whether using PODS during discharge was feasible and easy, whether it helped improve teaching efficiency, and whether it was helpful for patients and caregivers. Responses were collected on a five-point Likert scale (strongly disagree, somewhat disagree, neutral, somewhat agree, strongly agree).

The penetration rate of the intervention was assessed monthly by calculating the proportion of eligible patients who received the discharge summary based on clinical records.

Fidelity, including the completeness and quality of the PODS content, was assessed jointly by the research collaborator and the senior patient partner involved in the project. Copies of the completed PODS were made on paper by the research collaborator or the on-site project lead before patient discharge. Prior to the main assessment, each evaluator independently reviewed a small number of PODS copies to ensure a shared understanding and alignment in their subjective criteria. Following this calibration phase, all PODS were assessed in duplicate. Copies of the completed summaries were collected by the research collaborator before patient discharge and analyzed by the two evaluators. They independently rated each PODS on two dimensions: patient-centeredness and clarity and simplicity of language, using a 0–5 scale. The final scores were established through consensus discussions between the two raters.

Fidelity was also assessed through two questions included in the online feasibility survey, which asked healthcare professionals to estimate, over the past week, how many times they had used PODS and how often they had involved caregivers in the process, choosing from the following categories: 0–3 times, 4–6 times, 7–10 times, or more than 10 times.

Acceptability and appropriateness of the PODS for patients were evaluated through additional questions in the follow-up questionnaires for the intervention group. Participants reported ease of use, usefulness in preparing for discharge with the care team, continued utility at home, and willingness to recommend the PODS to others. Response options were “yes”, “no”, or “no opinion”, with space provided for open-ended comments.

### Data analysis

Standard statistical analyses were used to describe the data, that is, mean and standard deviation or median and interquartile range for continuous variables and absolute and relative frequencies for categorical data. Given the nature of the CTM-15 outcomes, linear regression was used to analyze the effect of the intervention on both the CTM-15 total score and the individual item-level scores. Although item-level responses are ordinal, we prioritized linear models for the primary analysis because their coefficients provide an intuitive and easily interpretable effect estimate. As a sensitivity analysis, we re-estimated all item-level models using ordered logistic regression, thereby altering the analytical assumption to reflect the ordinal scale of the data. This allowed us to assess whether our findings were robust to a modelling approach more strictly aligned with the data's measurement properties. The analyses were adjusted for a predefined (based on the literature) set of potential confounders, including educational background, living arrangement (cohabitation), recent hospitalizations, home care utilization, and functional status. Missing data were not imputed; therefore, analyses were conducted on an available-case population (i.e., complete-case analyses). Data were evaluated for outliers using standard descriptive statistics (percentiles) and visual inspection (histograms, scatterplots, and boxplots). No outliers were found. Differences between the two groups regarding post-discharge difficulties (ELADEB card-sorting results) were examined descriptively. We compared the frequency with which each domain was classified as a problem across groups and explored patterns in the distribution of reported difficulties. All data preparation and statistical analyses were performed using Stata Version 19 (22).

Qualitative data were drawn from the initial working group session, during which the CFIR card game was used, from two focus groups conducted with interprofessional team members, and from four interviews at the end of the implementation phase. These recordings were transcribed verbatim. A rapid directed content analysis was conducted, guided primarily by the CFIR 2.0 codebook (40, 41). The CFIR 2.0 provided the primary deductive structure for coding, and inductive codes were created when data did not fit the existing constructs. All transcripts were imported into MAXQDA software (23). Two researchers conducted the analysis: the principal investigator, a doctor in nursing science with extensive expertise in implementation research and in applying the CFIR framework, and a research collaborator, a specialized nurse clinician trained in qualitative methods. Prior to full analysis, they jointly coded a sample of transcripts to ensure consistency. They then coded the remaining transcripts independently, meeting regularly to compare interpretations, resolve discrepancies, and refine the coding scheme. Because each CFIR construct can function either as a barrier or a facilitator, we created additional codes to explicitly distinguish these two possibilities during analysis. This required revisiting earlier transcripts to classify each coded segment accordingly and to ensure that the distinction was analytically meaningful within the content analysis.

### Ethical considerations

The study was approved by the Cantonal Ethics Committee on Human Research of Vaud (2023-02048) in accordance with Swiss regulations and institutional guidelines for research involving human participants. For all recorded sessions— working group meetings, focus groups, or post-implementation interviews with healthcare professionals—written informed consent was obtained prior to participation.

## Results

### Intervention results

Of the 217 eligible patients invited to participate, 87 declined to participate, most often because they felt too occupied or overwhelmed by health problems, or because they anticipated minimal difficulties following discharge. A total of 66 participants were included in the control group and 64 in the intervention group (Supplementary Material S3). Among these, 11 participants were excluded from the control group (lost to follow-up, *n* = 5; change of discharge destination, *n* = 5; hospital readmission, *n* = 1) and eight from the intervention group (lost to follow-up, *n* = 5; change of discharge destination, *n* = 2; transferred to another department, *n* = 1). Data from 55 to 56 participants were included in the final analysis. Table 1 summarizes the participants' demographic and clinical characteristics.

**Table 1:** Participants’ characteristics.

	Control group *N* = 66	Intervention group *N* = 64
Gender		
Female, *n* (%)	36 (54)	27 (42)
Male, *n* (%)	30 (45)	37 (58)
Age, M (SD)	71.4 (11)	74.2 (9)
Min-Max	50–93	50–93
Education		
No certificate, *n* (%)	7 (11)	14 (23)
Middle/High school diploma, *n* (%)	9 (14)	5 (8)
Vocational Education and Training, *n* (%)	29 (47)	25 (40)
University degree *n* (%)	17 (27)	15 (24)
Living alone, *n* (%)	23 (35)	20 (31)
Home care utilization, *n* (%)	12 (18)	19 (30)
Previous hospitalization within the past 12 months, *n* (%)	20 (31)	28 (45)
Length of stay, M (SD)	8.1 (7.1)	8.8 (8.0)
Median (IQI)	6 (4–11)	7 (4–9)
Activities of daily living (1–6)	5.7 (0.9)	5.7 (1.0)
Main reasons for hospitalization *n* (%)		
Cardiovascular disorders	19 (29)	16 (25)
Pulmonary/ respiratory disorders	11 (17)	20 (31)
Neurological disorder	7 (11)	3 (5)
Nephrological/ urological disorders	4 (6)	7 (11)
Gastrointestinal/ hepatic disorder	7 (11)	3 (5)
Other	15 (27)	15 (23)
Categories of medication M (SD)		
Alimentary tract and metabolism	1.7 (1.3)	1.5 (1.3)
Blood and blood forming organs	0.8 (0.7)	0.8 (0.7)
Cardiovascular system	1.6 (1.5)	2.0 (1.6)
Dermatological	0.2 (0.5)	0.1 (0.4)
Genito urinary system and sex hormones	0.1 (0.2)	0.2 (0.4)
Systemic hormonal preparations, excl. sex hormones/insulins	0.2 (0.4)	0.3 (0.5)
Anti-infective for systemic use	0.3 (0.5)	0.3 (0.5)
Antineoplastic and immunomodulating agents	0.1 (0.2)	0.0 (0.2)
Musculo-skeletal system	0.2 (0.5)	0.3 (0.6)
Nervous system	1.4 (1.2)	1.0 (1.1)
Respiratory system	0.2 (0.5)	0.2 (0.4)
Sensory organs	0.0 (0.0)	0.0 (0.2)
Total number of ATC classes	7.3 (3)	7.2 (4)

M, mean; SD, standard deviation; Min, minimum; Max, maximum; IQI, interquartile interval; ATC, anatomical therapeutic chemical classification system. Length of stay is reported in days. Activities of daily living range from 1 (dependence) to 6 (independence). Percentages may not sum to 100 due to rounding.

#### Quality of care transition

A total of 55 (83%) participants in the control group and 56 (88%) participants in the intervention group completed the CTM-15. The evaluation of the perceived quality of care transitions revealed statistically significant differences between the groups, both for specific questionnaire items and for the overall score (Table 2). Patients in the intervention group reported significantly higher care transition experiences than those in the control group, as reflected in the total CTM-15 score [M = 74.4 vs. M = 62.3, *β* = 12.24, 95% CI (5.63, 18.85), *p* < 0.001]. At the item level, significant between-group differences were observed in several domains, including having a written and easily understood care plan (M = 3.1 vs. M = 1.9, *p* < 0.001), understanding of health condition (M = 3.4 vs. M = 3.0, *p* = 0.013), confidence in managing health (M = 3.6 vs. M = 3.2, *p* = 0.001), and understanding potential side effects of medications (M = 2.9 vs. M = 2.1, *p* = 0.004).

**Table 2:** Comparison of CTM-15 item and total scores between intervention and control groups.

CTM-15 items	Control group (*N* = 55) M (SD)	Intervention group (*N* = 56) M (SD)	B	95% CI	p	N
Before I left the hospital, the staff and I agreed about clear health goals for me and how these would be reached	3.3 (0.9)	3.2 (0.8)	0.04	[−0.28, 0.37]	0.789	101
The hospital staff took my preferences and those of my family or caregiver into account in deciding what my health care needs would be when I left the hospital.	3.4 (1.1)	3.5 (1.0)	0.26	[−0.13, 0.65]	0.185	94
The hospital staff took my preferences and those of my family or caregiver into account in deciding where my health care needs would be met when I left the hospital.	3.7 (1.1)	4.1 (1.1)	0.01	[−0.50, 0.51]	0.984	69
When I left the hospital, I had all the information I needed to be able to take care of myself	3.1 (0.7)	3.4 (0.8)	0.22	[−0.10, 0.55]	0.180	109
When I left the hospital, I clearly understood how to manage my health.	3.2 (0.6)	3.4 (0.6)	0.17	[−0.08, 0.43]	0.186	108
When I left the hospital, I clearly understood the warning signs and symptoms I should watch for to monitor my health condition.	3.1 (0.8)	3.4 (0.8)	0.30	[−0.00, 0.60]	0.050	105
When I left the hospital, I had a readable and easily understood written plan that described how all of my health care needs were going to be met.	1.9 (1.3)	3.1 (1.1)	1.07	[0.63, 1.50]	<0.001	102
When I left the hospital, I had a good understanding of my health condition and what makes it better or worse.	3.0 (0.9)	3.4 (0.8)	0.39	[0.08, 0.69]	0.013	102
When I left the hospital, I had a good understanding of the things I was responsible for in managing my health.	3.3 (0.9)	3.6 (0.7)	0.31	[0.03, 0.59]	0.030	99
When I left the hospital, I was confident that I knew what to do to manage my health.	3.2 (0.9)	3.6 (0.7)	0.45	[0.18, 0.73]	0.001	102
When I left the hospital, I was confident I could actually do the things I needed to do to take care of my health.	3.2 (0.8)	3.5 (0.7)	0.40	[0.10, 0.71]	0.010	101
When I left the hospital, I had a readable and easily understood written list of the appointments or tests I needed to complete within the next several weeks.	2.3 (1.3)	3.1 (1.3)	0.62	[0.09, 1.15]	0.022	100
When I left the hospital, I clearly understood the purpose for taking each of my medications.	3.4 (0.8)	3.6 (0.7)	0.28	[−0.00, 0.57]	0.054	105
When I left the hospital, I clearly understood how to take each of my medications, including how much I should take and when.	3.4 (0.8)	3.6 (0.8)	0.19	[−0.11, 0.49]	0.203	107
When I left the hospital, I clearly understood the possible side effects of each of my medications.	2.1 (1.2)	2.9 (1.2)	0.64	[0.21, 1.07]	0.004	101
Total score (0–100)	62.3 (15.3)	74.4 (15.3)	12.24	[5.63, 18.85]	<0.001	111

M, mean; SD, standard deviation; B, coefficient; CI, confidence interval; *p*, *p*-value; N, number of participants. Models were adjusted for education, cohabitation, hospitalizations, home care, and activities of daily living (ADL) score.

Other items showed a positive but non-significant trend, such as understanding warning signs (*p* = 0.050) and understanding the purpose of medication (*p* = 0.054). No meaningful group differences were observed for items related to agreement on health goals or consideration of patient and family preferences (*p* > 0.18).

#### Post-discharge difficulties

Among the participants who completed the CTM-15, 22 in the control group (40%) and 23 in the intervention group (41%) also took part in the ELADEB card-sorting exercise. No statistical differences were observed between the groups. Across both groups, difficulties most frequently concerned physical condition (reported by 73% in the control group and 91% in the intervention group), mental health (36% vs. 44%), food (36% vs. 39%), and medication (32% vs. 35%). Transportation issues were slightly more common in the control group (50%) than in the intervention group (39%). Supplementary Material S4 provides the complete distribution of reported difficulties across the 20 life domains.

### Implementation results

#### Key implementation determinants

To inform the implementation process, key determinants and initial strategies were developed based on four workshops with the working group, two focus groups with unit professionals (*n* = 6, *n* = 10), and results from the NoMAD questionnaire (*n* = 30). The 30 participants who completed the NOMAD questionnaire had an average age of 34.6 years (SD = 10.8), were mainly nurses (40%), followed by physicians (20%). Nursing assistants and physiotherapists each represented 13.3% of the sample, while auxiliary nurse accounted for 10% and dietitians for 3.3%. Regarding overall professional experience, 43.3% had more than 10 years of experience, while 26.7% had between three and five years and 20% reported less than two years of experience. Experience within the current unit was fairly balanced, with 23.1% having less than one year of experience, 57.7% one to five years, and 19.2% more than five years. Results of the NoMAD are presented in Supplementary File S5.

The identified barriers and facilitators are presented below according to the CFIR domains.

##### Innovation

Co-developed by patients and healthcare professionals, PODS was valued for its personalization, clarity, and support for discharge teaching. In the feasibility survey, 70% of the participants recognized its value for discharge teaching, and 85% recognized its value for patients and caregivers.

“It's written down, which is good because there's a lot of information given orally. The fact that it's written is great”. (physiotherapist, focus group 2)

Adaptability, appealing design, and low cost facilitated their acceptance. This was considered especially relevant for certain clinical contexts (e.g., cognitive impairment and complex discharge). While 65% of the participants rated the tool as easy to use in the feasibility survey, collaborative and interprofessional completion with patients was more complex due to unclear role distribution.

“ I wouldn’t say it's complex, but as we see, it doesn’t seem too complex. Then again, yes, it involves several providers. It's always about coordinating with everyone to get this document completed”. (nurse, working group 1)

##### Inner setting

High workload, frequent patient turnover, and chronic understaffing—especially among medical staff, dietitians, and liaison nurses—were consistently reported as major barriers, limiting the time and capacity to complete the PODS in an already resource-constrained environment. About one third (37%) of the 30 NoMAD respondents agreed that adequate resources were available for the project. Compatibility with existing workflows was mixed; while the medical visit offered an opportunity to introduce the tool, completion was anticipated to be particularly difficult for unplanned discharges or very short stays. Physiotherapists' frequent rotations, lack of attachment to the unit, and non-utilization of the EHR system further complicated their involvement. Facilitators included the planned transition to EHR, which could support the integration of the PODS in the workflow, and accessible physical placement at the bedside. However, the strong medical delegation culture of the units limited nurses' autonomy in discharge planning.

##### Individuals

The analysis considered individuals involved in PODS implementation (working group), delivering the intervention (unit team), and receiving it (patients). The nursing directorate provided strong initial support, legitimizing the project within the institution, but this was not yet fully visible to the team prior to implementation—40% of NoMAD respondents agreed that management supported the PODS, while 27% were neutral. The unit team generally showed motivation towards change: 77% of NoMAD respondents said they would support the PODS. Capability was also considered relatively strong, with 77% confident in colleagues' ability to use the tool, and all professionals considered able to support patients in completing it. However, the written nature of the tool raised concerns in the working group, especially for complex topics or fear of making errors. Newly graduated nurses often did not consider patient education as part of their autonomous role. Opportunities were perceived as insufficient: lack of time and suboptimal discharge organization were anticipated to limit consistent and meaningful use, which over time could reduce motivation. The PODS was recognized by the working group as meeting patients’ and caregivers' needs: patients are often discharged with minimal information and report confusion or forgetfulness regarding verbal instructions. However, according to the working group, these needs may depend on patients' health conditions and autonomy. Patient capability to engage with the PODS was anticipated to be variable, with some requiring writing support and others facing language barriers or cognitive impairment.

#### From contextual determinants to implementation strategies

Table 3 provides an overview of all implementation strategies mobilized for the PODS intervention, categorized by type, actors, and timing. A first set of strategies was developed in response to the determinants of implementation identified in the preceding context analysis (T0). For example, several adaptations were deemed necessary to ensure the PODS would be implementable in the unit. Multiple rounds of team discussions and consensus-building led to adjustments of both the content and the visual layout, with a patient partner playing a central role in refining wording and format. The hospital communication service was subsequently engaged to redesign the graphic layout in line with the institution's visual identity and to take charge of printing, thereby enhancing sustainability. Another major focus concerned training: although the tool itself was considered simple to use, unit leadership emphasized the importance of a durable support to maintain the intervention over time and to orient new staff. In response, the working group co-developed a short training video, featuring two nurses from the unit to reinforce local ownership. The video was initially circulated by email and later made permanently available on the hospital intranet. Practical issues were also considered, ranging from the placement of PODS in patient rooms to its integration into the electronic health record, daily monitoring sheets, and electronic screens in staff offices.

**Table 3:** Overview of implementation strategies according to the ERIC taxonomy, responsible actors, and timing.

Type of strategy	Actions	Research team	Hospital/unit team	T0	T1
Use financial strategies	Fund the research project and allocate a hospital contribution to release staff time so collaborators can actively participate in the implementation process	X		X	
	Fund and manage the printing of PODS copies through the hospital communication service		X	X	
Engage consumers/users	Involve a patient partner for visual adjustments of the PODS and adapt the content section with the feedback from the team	X		X	
	Prepare patients to be active participants by producing a patient information video in collaboration with the patient partner. The QR code linking to the video was displayed on posters in the corridors and printed on the back of the PODS.	X		X	
	Develop and distribute an additional flyer to patients/caregivers to further support their understanding and use of the PODS.	X			X
Use evaluative and iterative strategies	Evaluate and prioritize the most important determinants (CFIR card game, focus groups, NoMad questionnaire)	X		X	
	Develop an implementation blueprint detailing all steps of the pre-implementation and implementation phases, including the timeline, assigned responsibilities, and expected outputs.	X		X	
	Monitor implementation and intervention outcomes and regularly share the results with the working group to determine whether adaptation of the strategies was needed.	X			X
Develop stakeholder relationships	Develop academic partnerships: an existing partnership between the research team and the hospital provided the opportunity to integrate expertise in implementation science and effectiveness research into the project.	X		X	
	Establish an interprofessional working group within the unit, including a designated on-site project lead		X	X	
	Organize regular working group meetings to prepare and monitor the implementation process	X	X	X	X
	Identify and prepare clinical nurse specialists to support the PODS integration in the unit		X	X	
	Identify early adopters in the unit team who perform the PODS intervention particularly well		X		X
	Cultivate relationships with the working group by maintaining regular communication and ongoing exchanges.	X		X	X
	Use media to share information about the project through the hospital intranet, LinkedIn, and presentations at internal professional meetings.	X	X	X	X
Provide interactive assistance	Provide supervision and assistance to the unit team by the clinical nurse specialists and the on-site project lead		X		X
Support clinicians	Remind the team to use the PODS through different means, such as prompts during morning interdisciplinary meetings, reinforcement by clinical nurse specialists throughout the day, visual cues on posters in staff offices, and electronic prompts in the patient record.		X		X
Train and educate stakeholders	Develop and distribute educational materials: training video with nurses from the unit team, examples of pre-filled PODS, and posters explaining how to correctly complete the PODS.	X		X	
	Integrate the training video into the onboarding process for new staff members.		X		X
Change infrastructure	Mandate the PODS implementation as a priority project for the unit by the nursing department leadership, demonstrating their determination to implement it		X	X	
	Make adjustments to the EHR to facilitate integration of the PODS within routine workflows		X	X	
	Identify on the electronic screens in the offices, as well as on the daily monitoring sheets, the patients who received the PODS.		X	X	
	Equip patient rooms with whiteboards to display the PODS summaries using magnets, ensuring they are visible to both patients and healthcare staff.		X		X
Adapt and tailor to context	Promote adaptability of the PODS by adjusting the visuals and the content	X		X	
	Tailor strategies to address initial and emerging barriers and leverage facilitators through regular working group meetings		X	X	X
	Integrate the PODS into the clinical workflow by embedding it in existing processes and tools (e.g., discharge checklist in the EHR, admission pack, daily monitoring sheets, morning multidisciplinary meetings, and follow-up during nursing and medical visits).		X		X

T0 = prior to implementation start; T1 = during implementation.

#### Context monitoring

Monitoring of the implementation determinants was conducted through regular working group meetings, field observations, and feasibility surveys, enabling iterative adjustments to address emerging challenges. Despite implementation progress, several practical challenges persisted, including omissions of key information and lack of follow-up entries in the EHR, difficulty ensuring continuity after PODS initiation, unclear weekend organization, and completion of PODS sections often missed early on. Physicians tended to provide discharge recommendations close to the actual discharge time, which delayed documentation. The physical placement of the PODS in patients' rooms was sometimes problematic, leading to misplaced documents and a lack of visibility. Engagement varied across the professional groups. Physiotherapists faced structural constraints but showed increased alignment over time by checking whiteboards in nurses' office to identify PODS use. Dietitians had limited opportunities due to the timing of their interventions but introduced workarounds, such as patient flagging. Liaison nurses often forgot to use the tool and perceived it as overlapping with their existing tasks. Physicians acknowledged its value for communication but questioned systematic use due to resource constraints. Reduced interdisciplinary engagement and limited perceived value in some groups contributed to declining motivation among the nursing team.

External factors also disrupted the process: an influenza outbreak reduced team availability and completion rates, and the end of data collection reduced on-site support and removed a reinforcement factor.

As implementation unfolded, the emerging barriers led to the development and adjustment of additional strategies (T1) (Table 3). When adoption was uneven, reminders and supervision by clinical nurse specialists were reinforced to support consistent use of PODS. Practical adjustments were also made after it became clear that PODS stored in bedside tables were often misplaced or overlooked; in response, whiteboards were installed in patient rooms to display the PODS more visibly and accessibly. Further training resources were created, such as examples of pre-filled PODS, to provide teams with clearer guidance on the type of content expected. Finally, one of the most effective strategies proved to be simply allowing time for the intervention to become embedded in routine practice, enabling staff to gradually integrate PODS into their daily workflows.

#### Persistent determinants

At the end of the implementation phase, four interviews with healthcare professionals (two nurses, one nurse clinician, and one physiotherapist) contributed to identifying persistent obstacles to PODS implementation. A key barrier was the perceived misalignment between the tool and the work organization. Heavy workload, frequent short stays, and the fast pace of care limited integration: “*We only have half an hour, file included. So, it's quite tightly timed*”. (Physiotherapist). Short stays sometimes meant the PODS was given but not completed: “*Several times they left before we had the time to fill it out with them*”. (Nurse 1). Limited anticipation of discharge planning further compounded these challenges.

Perceived usefulness strongly influenced engagement: “*If it has meaning, I’ve seen that practices happen much more smoothly*”. (Physiotherapist). Feedback on patient benefit was seen as a motivator, though benefits were often only visible after discharge: “*The benefits aren’t seen during the stay*”. (Clinical nurse specialist). Professionals stressed the importance of clearly communicating the tool's value to patients and the staff.

PODS was valued for supporting understanding, self-management, and clarity, especially for new diagnoses or chronic conditions, and for fostering trust: “*It really helps to seal that therapeutic alliance with the patient*”. (Nurse 2). For some, the interaction around the tool was as valuable as the tool itself: “*We make something tangible*”. (Nurse 2). However, patients' ability to use it varied, with cognitive impairments, language barriers, and low motivation posing challenges to its use. Some professionals doubted its use in the home setting.

A lack of interprofessional involvement persisted, with nurses often bearing responsibility alone: “*In practice, it was very rarely filled out by other professionals*”. (Clinical nurse specialist). Champions such as nurse clinicians and the head nurse played a key role in reminders and patient identification. Yet, the process lacked automation, motivation waned over time, and turnover hindered consistent use: “*There's a lot of turnover* *…* *colleagues ask, ‘I’ve never done it, I don’t know how to do it*’”*.* (Clinical nurse specialist).

Concrete aids, particularly pre-filled examples, were widely appreciated: “*The examples really helped colleagues in practice*”*.* (Clinical nurse specialist), helping to reduce uncertainty about what and how to write.

### Implementation outcomes

#### Feasibility

Supplementary Material S6 presents the results of the survey conducted among the healthcare team at three different time points (T1: start of implementation (*n* = 20), T2: after three months of using the discharge summary (*n* = 15), and T3: after five months (*n* = 24). Regarding workload, 85% of professionals at T1 and T2 reported that the discharge summary increased their workload (*n* = 17; *n* = 13). At T3, this perception decreased, with 58% (*n* = 14) still expressing this view, along with an increase in the number of professionals who disagreed (*n* = 6; 25%).

The feasibility of using the summary was considered favorable by 65% of the professionals at T1 and T2 (*n* = 13; *n* = 10). This rate decreased to 54% (*n* = 13) at T3, with an increase in negative responses (*n* = 4; 17%).

Ease of use was positively rated by 65% of professionals at the first two points in the study (*n* = 13; *n* = 10). A marked improvement was observed at T3, reaching 83%, reflecting the increased familiarity and adoption of the tool by professionals.

Perceived effectiveness of discharge teaching was initially well received, with 70% agreement at T1 and T2 (*n* = 14; *n* = 11). At T3, the perception remained positive (*n* = 16; 67%). However, the distribution of agreement levels shifted, indicating a slight reduction in the proportion of strong agreement compared to earlier assessments.

Finally, the usefulness of the discharge summary for patients and caregivers was strongly recognized in the first two assessments (*n* = 17; *n* = 13; 85%). This proportion remained high at T3 (*n* = 17; 71%), despite an increase in neutral responses, indicating a more nuanced evaluation of the perceived impact.

#### Fidelity

Among the 56 participants in the intervention group, all received the PODS. However, due to various circumstances—such as unexpected or accelerated discharges—only 37 PODS (66%) contained at least one completed section. Completion rates for individual sections varied (Supplementary Material S7). The most frequently completed components were reason for hospitalization (89%), current health issues (81%), and symptoms (70%). In contrast, more action-oriented sections, such as what to do (41%) and warning signs (54%), were completed less consistently. Regarding lifestyle-related information, type of activity (59%) and instructions (43%) showed moderate completion rates. Sections related to where to find more information were among the least completed, with only 35% including the name of a contact person and 22% providing contact details. The evaluation of the PODS content indicated a high level of patient-centered care. On a scale from 0 to 5, the mean score for patient-centeredness was 4.9, suggesting that the content was highly aligned with patients' needs. The clarity and simplicity of the language used in the PODS were rated positively, with a mean score of 4.7. These results indicate that the content was generally easy to understand and accessible to the intended patients.

Fidelity was also explored through two survey questions regarding specific delivery practices. Across all three time points, most professionals reported including caregivers in discharge teaching using the PODS on average 0–3 times per week (78% at T1, 93% at T2, 92% at T3), with very few reporting higher frequencies of use. Similarly, the teach-back technique was most often used 0–3 times (72%, 100%, and 83% at T1, T2, and T3, respectively). Only a small proportion reported more frequent use—4–6 times (17% at T1 and T3) or 7–10 times (11% at T1, none at later time points). These results suggest limited systematic use of these key components of the intervention.

#### Penetration

In the first month of implementation, the penetration rate was 36% (*n* = 105 eligible patients). This rate increased to 44% in December (*n* = 88), followed by a slight decrease to 36% in January (*n* = 97), likely due to a seasonal influenza outbreak during that period. In February, a significant increase was observed, with the penetration rate reaching 61% (*n* = 85).

#### Acceptability and appropriateness for patients

Most participants (*n* = 39; 72%) found the tool relatively easy to read and use, although several noted that filling it out without assistance was challenging: “*It was easy to read, but difficult to fill out without help*”. Some participants were initially confused: “*I didn’t understand what to write, but the nurse helped me afterwards*” (Table 4).

**Table 4:** Perceived appropriateness and acceptability of the patient-oriented discharge summary (*N* = 54).

Questionnaire items	Yes *n* (%)	No *n* (%)	No opinion *n* (%)
Was the PODS easy to use?	39 (72)	7 (13)	8 (15)
Was the PODS helpful in preparing your discharge home with the team?	24 (44)	20 (37)	10 (19)
Was the PODS useful once back home?	16 (30)	29 (54)	9 (17)
Would you recommend the PODS to other patients?	43 (80)	4 (7)	7 (13)

Experiences about the usefulness to prepare the hospital discharge varied, 24 participants (44%) found it helpful for reflecting on their needs and for preparing conversations with healthcare providers, others, especially those who felt more autonomous, said it added little: “*It helped me ask questions I wouldn’t have thought of before discharge*”; “*I already knew everything that was written* *…* *it didn’t add much*”.

Some participants (*n* = 16; 30%) consulted the PODS to monitor symptoms, but others felt it was unnecessary, as they had already received verbal instructions or found it too vague. The majority of participants (*n* = 43; 80%) would recommend the tool, especially for patients living alone, very old adults and those who might struggle with organizing information post-discharge: “*It's a good tool to organize the discharge* *…* *useful for people who forget things*”; “*It's important to not throw people out without guidance* *…* *especially for older adults*”*.*

## Discussion

This study evaluated the implementation and impact of a patient-oriented discharge summary on the perceived quality of care transition among hospitalized adults in Switzerland. The intervention was associated with significantly higher CTM-15 scores, reflecting improved preparedness for self-care, clearer understanding of responsibilities and health conditions, better awareness of medication side effects, and more frequent receipt of written follow-up instructions. However, despite these improvements, the PODS did not reduce the type or frequency of post-discharge problems reported by patients.

### Challenges of using a collaborative blank version of PODS

Unlike the pre-filled versions tested elsewhere (26–28), a distinctive feature of our study was the use of a collaborative blank version of the PODS, completed progressively with patients. This format fostered individualized teaching and patient engagement, but it also contributed to variability in completion, potentially reducing the tool's usefulness once patients returned home. This design choice is key to interpreting our findings: PODS proved effective in structuring discharge communication and fostering a sense of preparedness but was less effective in providing concrete post-discharge guidance. The blank format encouraged personalization but sections requiring detailed instructions—such as warning signs or follow-up actions—were often incomplete. Similar to our study, Hahn-Goldberg et al. (24) and Okrainec et al. (25) tested a blank collaboratively completed version of the PODS. However, unlike our findings, the results did not show statistically significant improvements in patients' understanding of medications or in their knowledge of what to do if problems arose after discharge. These findings contrast with studies evaluating pre-filled or standardized PODS versions, which tend to ensure completeness and usability at home, but may offer fewer opportunities for co-production during hospitalization. For example, Black et al. (26) reported that 88% of patients found the PODS at least partly helpful, 79% felt adequately prepared for discharge, and 98% of patients and families believed that the PODS added value to the discharge experience. Similarly, Schofield et al. (27) tested a heart failure–specific adaptation of the PODS, which included pre-filled standard information. This version was associated with higher rates of recall of written information, more frequent use of the materials as visual reminders, and improved adherence to dietary and physical activity recommendations after 30 days (27). However, in that study, patients often did not consult the materials during the initial days post-discharge, and individual teaching provided by ward staff was identified as the most influential factor in behavior change (27). An adaptation of the PODS for intensive care also showed positive results, particularly in terms of patients' understanding the medical condition (28). Additionally, 80% reported having a conversation with the ICU team about the transition and the next steps (28). Similarly, a comparative study in Ontario found that hospitals with full PODS implementation had higher odds of patients reporting discussions with staff about the needed help and receiving written information on post-discharge symptoms (25). Future adaptations could therefore combine standardized pre-filled content for core instructions with individualized sections completed collaboratively, ensuring that both informational accuracy and patient engagement are maintained.

Our findings emphasize the relational value of the PODS. Patients reported limited use of the tool after discharge but consistently highlighted the usefulness of the conversations it prompted with healthcare professionals. These findings suggest that the PODS is effective less as a written document than as a structure that supports communication, shared understanding and active participation. Consequently, its value emerges mainly during hospitalization, where it acts as a relational and cognitive scaffold rather than a static information tool. This raises broader questions about the role of discharge tools: their main contribution may lie in structuring and enhancing teaching and dialogue during hospitalization, rather than to sustaining self-management behaviors once patients are at home. A literature review by Okrainec et al. (29) supports this interpretation, showing that patient-centered discharge tools improve comprehension but do not consistently translate into better adherence or outcomes (29). Similarly, previous studies have not demonstrated that PODS reduces unplanned healthcare utilization or hospital readmissions (24, 30).

This relational function reflects the core purpose of the PODS—a tool designed to guide discharge teaching and synthetize key information for the post-hospital period. Healthcare professionals repeatedly emphasized this pedagogical role, describing the PODS as “*a structure that helps us not forget the essentials*” and “*a support for the conversation rather than a document to fill*”. In surveys, 70% of respondents recognized its value for discharge teaching, and 85% rated it as helpful for patients and caregivers. Together, these findings confirm that the PODS primarily serves to organize and clarify information during discharge preparation. However, patients still face numerous practical challenges beyond what the tool covers, such as transportation difficulties or limitations in daily activities. These domains fall outside the scope of the PODS, which focuses mainly on medical and self-care information. Consequently, while it enhances comprehension of clinical instructions, it does not address broader determinants of a successful transition—such as mobility, social support, or community resources. This may help explain why improved perceptions of care transition quality did not translate into fewer post-discharge problems: many of these difficulties stem from contextual and social barriers rather than from informational gaps.

Our findings suggest that this tool alone may not be sufficient to address these issues. The use of blank PODS forms completed collaboratively with patients, rather than pre-filled versions with standardized content, may have limited the consistency and completeness of the information recorded. Some participants likely received the PODS with minimal content, which may not have provided adequate guidance for managing symptoms and other post-discharge challenges. The predominance of physical health problems among reported difficulties further highlights the complexity of post-hospital recovery, which extends beyond information provision. Effective transitional support therefore requires multifaceted strategies that combine discharge teaching with ongoing clinical, social and community-based support to better address patients' diverse needs once home.

### Linking implementation to outcomes

In addition to providing evidence on the effects of PODS for patients, this study highlights how implementation determinants shape clinical outcomes. Workload constraints, limited workflow compatibility, and variable interprofessional engagement influenced PODS use. For example, incomplete sections of the tool reflected both time pressure and inconsistent ownership across professional groups, limiting the guidance that patients received at home. Fidelity analysis confirmed this: while general sections, such as the reason for hospitalization, were usually completed, more action-oriented components (e.g., what to do, warning signs, or contact details) were often omitted. As a result, the information received by PODS patients was personalized and clear, but not always comprehensive enough to support symptom management after discharge. This helps explain why patients felt better prepared in the hospital but still reported similar post-discharge problems.

Concerns about workload were common at the start of implementation but decreased as staff gained experience, echoing findings by Black et al. and Hahn-Goldberg et al. (24, 26). They also both reported that greater experience with PODS increased professionals' confidence that patients were prepared for discharge. We did not assess this quantitatively, but interviews in our study revealed that while some staff perceived PODS as improving patient readiness, others doubted whether patients would use it at home. Thus, the impact of PODS depended not only on the intervention itself but also on its appropriation by the staff. Compared to pre-filled formats, which guarantee a minimum information set, blank forms may be more sensitive to workload and time constraints, but they also better support personalized, discussion-based teaching in our context (38).

Another key factor was uneven engagement across professional groups. Although designed as an interprofessional tool, PODS was mostly championed and completed by nurses, while other professionals engaged inconsistently due to workflow barriers or limited perceived value. This nursing-led appropriation limited the PODS capacity to integrate broader interprofessional contributions that might have strengthened continuity of care. Similar challenges have been reported in previous studies, in which sustained engagement across disciplines proved difficult. Black et al. (26) showed that visible, consistent engagement from unit leaders and clinical champions were critical for normalizing PODS use by reinforcing it during daily rounds, team huddles, and staff onboarding. In our setting, the working group noticed that when the nurse manager and clinical nurses actively reminded staff and monitored EHR entries, penetration rates increased, and motivation was sustained. Conversely, disruptions, such as the influenza outbreak, coincided with drops in completion rates and staff engagement. This suggests that leadership and continuous reinforcement are not only facilitators of implementation but also determinants of the consistency and quality of patient outcomes. Additional strategies might also have supported broader interprofessional engagement, such as regularly clarifying role expectations for each profession, offering periodic profession-specific feedback, highlighting interprofessional success stories, securing stronger visible endorsement from hospital leadership, explicitly linking the project to other institutional goals, and identifying influential clinicians as informal ambassadors. These options were beyond the scope of the current project but could be explored in future implementation.

Patient feedback can help interpret these patterns. Patients valued the discussions prompted by PODS, even when they did not use the document itself after discharge. This highlights that PODS' primary contribution may lie in its relational function—structuring discharge conversations and supporting teaching—rather than in its role as a written discharge summary. Where the PODS was used interactively—through a supportive dialogue between healthcare professionals and the patient—patients may have felt more prepared. Conversely, when completion was fragmented or rushed, the relational component of the interaction was reduced, which may have weakened its impact once the patient returned home. However, the blank format of the tool requires patients to actively document their information. Feedback from both patients and professionals indicated that some were not interested in or did not see the purpose of this exercise, raising broader questions about expectations regarding patient engagement in discharge preparation. In the acute hospital context, fatigue, the impact of illness, and—for older adults—the additional effects of aging may limit patients' readiness to engage with such a tool. This cultural aspect may also differ across contexts: in Switzerland, encouraging patients to co-produce their discharge summary represents a relatively new expectation, whereas in Canada PODS has been more widely sustained in some regions (24, 25). Integrating PODS into electronic health records could make the form easier for staff to complete. Some parts could be automatically filled in with standard information, while leaving space for healthcare professionals to add details specific to each patient. However, this should not replace the face-to-face explanations and discussions at discharge, which patients find particularly helpful.

### Strength and limitations

The use of a mixed-methods approach, combining surveys, focus groups, interviews, and field observations, provided a comprehensive understanding of the PODS implementation process in the study. The implementation was guided by the CFIR, which was used at baseline for the contextual analysis and throughout the monitoring phase. This structured approach helped identify key determinants, guided the selection and adaptation of strategies, and ensured systematic analysis. Using the CFIR also strengthens the transferability of our findings by enabling comparison with other international studies applying the CFIR, as it is the most widely used determinant framework. The study was conducted only in a single unit of one hospital, but testing the implementation under real-world acute care conditions, with healthcare team members, and within a setting characterized by organizational constraints strengthens the transferability of the results to other hospital environments. Several methodological limitations should be acknowledged. First, the study used a pre–post design with two groups but without randomization, which limits causal inference about the effects of the PODS on patient outcomes. In our study, fidelity was indirectly assessed through completion rates, which provided only partial insight. The absence of a clear trend in post-discharge problems may be explained by marked variability in PODS completion, with some forms fully completed, others partially completed, and others left largely blank. Completing the CTM-15 and assessing post-discharge problems 5–10 days after discharge may be subject to recall bias, as patients might have forgotten some of the difficulties faced in the first days at home or no longer perceived them as problems—either because they had adapted or adjusted their expectations. Finally, not all professional groups involved in discharge processes were represented in the qualitative components of the study. In particular, some professions—including physicians—did not participate in the post-implementation interviews nor in all working-group workshops, mainly due to workload and scheduling constraints. This limits the diversity of perspectives captured regarding the implementation, especially given the central role of certain professional groups in producing discharge summaries.

## Conclusion

This study shows that the Patient-Oriented Discharge Summary (PODS) improves older adults' experience of care transitions in a Swiss acute care setting, primarily by supporting more structured and meaningful discharge teaching. However, its impact after discharge was limited by contextual and organizational constraints, including variability in how the collaboratively completed form was filled out. Future adaptations—such as hybrid formats with pre-filled core elements integrated into electronic records—may enhance consistency while preserving relational value. Overall, PODS is a helpful but not sufficient standalone intervention. To optimise transitional care, it should be embedded within broader strategies that include post-discharge follow-up. Ultimately, sustained leadership engagement, interprofessional ownership, and a cultural shift towards active patient participation will be critical for embedding PODS in routine practice and maximizing its long-term contribution to patient-centered transitional care.

## Data Availability

The raw data supporting the conclusions of this article will be made available by the authors, without undue reservation.
