# Supplementary material for: Implementing a patient-oriented discharge summary to improve hospital-to-home transitions in older adults: lessons from a hybrid study
Source: Front Health Serv. 2026 Jan 16;5:1730127. doi: 10.3389/frhs.2025.1730127 (PMC12855460; doi:10.3389/frhs.2025.1730127)
Supplement: Supplementary file 1 [file Table1.docx]

# **Supplementary material 1:** The patient-oriented discharge summary (French version)


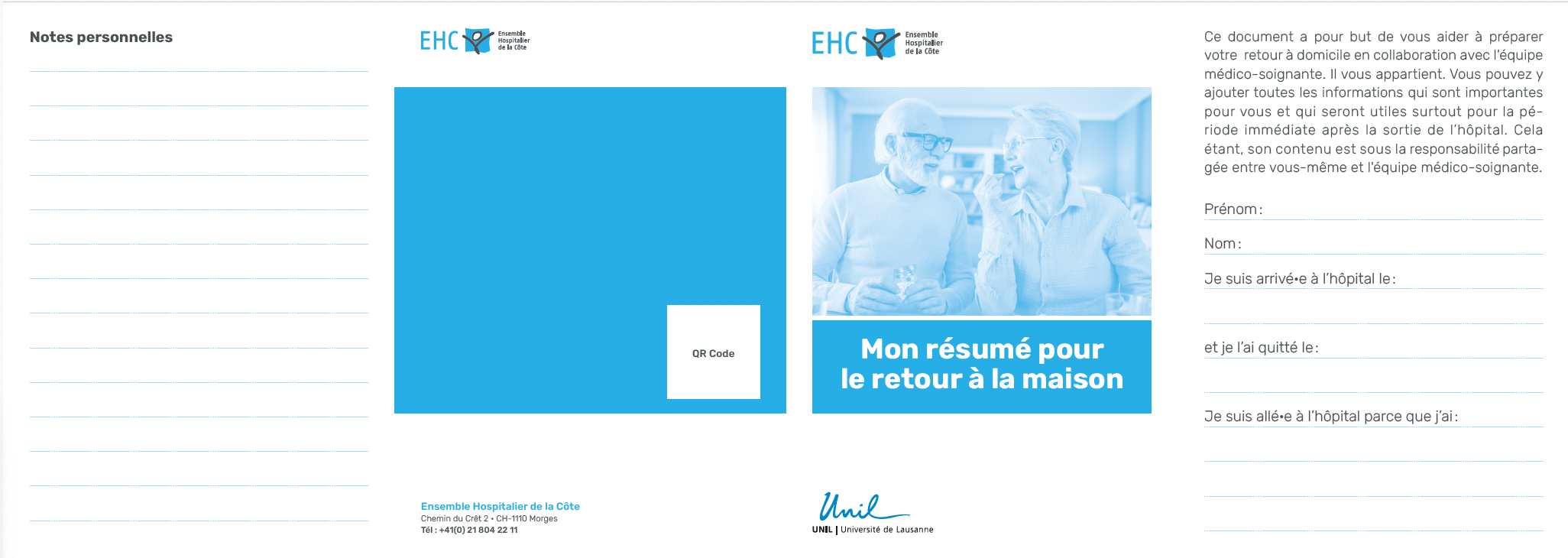


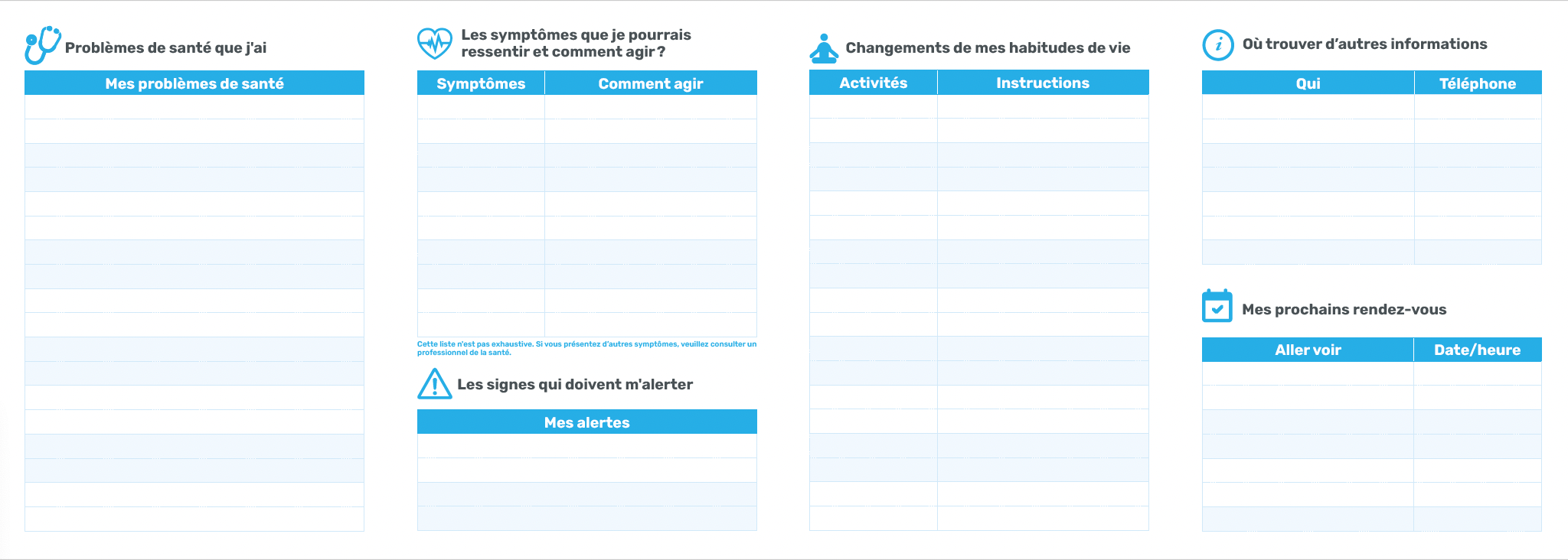


**Supplementary material 2. Interview and focus group guides**

**Focus Group Grid – Healthcare Professionals**

**Project title:** Improving the Hospital-to-Home Transition with a Patients Discharge Summary Tool for older adults.

**Date:** 08.04.2024 and 15.02.2024

**Professionals represented:** Physicians, physiotherapists, registered nurses, liaison nurses, nursing assistants, and dietitians.

**Focus group facilitator/moderator:** Dr.Sc. Joanie Pellet (investigator)

**Focus group observer:** Raquel Solano (research collaborator)

| **Time** | **Section** | **Thematic Areas and Interview Guide** | **Follow-up questions** |
| --- | --- | --- | --- |
| 5 min | Introduction | **Introductions and welcome** Begin by introducing yourself and thanking the participants for being here today.  **Study reminder** This project aims to implement a hospital Discharge Summary (PODS) for patients and their family caregivers, with the objective of facilitating the transition from hospital to home. This tool was originally developed in Canada with the active involvement of patients and relatives. It appears to address several needs related to information provision and the prioritization of essential instructions for the post-hospital period.  However, its adaptation to our local context, its effectiveness, and the way it can be integrated into current clinical practice still need to be explored. The project is therefore structured around two main objectives: first, to determine how the PODS can be concretely integrated into our existing discharge-preparation routines, complementing current practices; and second, to evaluate its actual impact on patients’ experience and satisfaction.  This is where the IUFRS team plays a role, by assessing the perceived benefits for patients and family caregivers using several evaluative measures. In parallel, we would like to gather the interprofessional team’s perspectives on the elements that may facilitate or hinder the use of such a document in everyday practice.  **Purpose and structure of the focus group** … And this is precisely the purpose of today’s session: to identify your perceptions regarding the challenges and facilitating factors you anticipate if you were to start using the PODS in your day-to-day work. We have 45 minutes, and the goal is to engage in a dynamic discussion centered around a few guiding questions.  **Confidentiality reminder and consent for audio recording** Before we begin, I want to emphasize that everything discussed within this group will remain confidential. We plan to audio-record the session for the sole purpose of transcription and subsequent analysis.  **Are you comfortable with this?**  **Questions?** Before we begin, do you have any questions? |  |
| 10 min | **Icebreaker question** | **Briefly present the process proposed by the working group to introduce the icebreaker question:**  The patient receives the PODS upon admission (within 24 hours). The PODS remains in the patient’s room and is completed throughout the hospital stay by the patient, with support from healthcare professionals depending on the section. Family caregivers are included whenever possible during teaching moments or informed about the PODS. Prior to discharge (24–48 hours), the remaining information is completed, and physicians add the treatment plan and upcoming appointments. At discharge, the patient goes home with the PODS.  **Icebreaker question:**  If we were to start using the PODS in practice starting tomorrow, what aspects might raise questions?  What do you think will make its use easy or difficult in general? | **Why do you think** X **might limit or facilitate the use of the PODS?**  **Could you elaborate on what you mean by?**  **Could you explain that a bit further?**  **Could you give an example?**  **To bring the discussion back to the group:**   - That’s interesting — does anyone have a similar or different perspective? - I’d like to hear how this resonates with the rest of you. |
| 10 min | Questions about advantages and limitations | **What do you see as the main advantage of using a tool like this for patients—and, conversely, its limitations?**  **What do you see as the main advantages of using a tool like this for you in your clinical practice, specifically when preparing patients for discharge—and, conversely, its limitations?** |  |
| 15 min | Questions about feasibility, organizational compatibility, and implementation conditions | **To what extent do you think the PODS is compatible with your care processes/your organizational workflow? How do you think the PODS could/should be integrated into your current care organization?**  **In which situations, or for which patients, do you think it might not be suitable or usable?**  **In what ways do you think the transition to an electronic medical record could be helpful, or conversely, could represent a limitation?**  **How do you think the team, your colleagues, will react to this project/this intervention?**  **To what extent is improving discharge preparation a priority for your unit?**  **What are your expectations for the working group for this project?** | **What role should each person have?**  **What strategies could encourage everyone’s collaboration around the PODS?**  **How does communication work within the team? And with the unit managers?**  What kind of support do you think is needed?  What should they put in place? How should they facilitate the implementation of the PODS?  **Based on your experiences with previous projects, what are the key things that should be done—or avoided—to ensure the success of this project?** |
| 5 min | Focus Group Closure | If you had to start using the PODS tomorrow, what is the one most important thing you would need to do?  **Introduce the closing section of the discussion:** We have X minutes left. I will briefly summarize what has been discussed and check with you whether this accurately reflects what you expressed. Please let me know if anyone would like to add anything before we conclude.  Thank you for participating. The data collected will be analyzed and used exclusively for the purposes of this study. |  |

**Structured Interview Guide for Professionals**

Project title: Improving the Hospital-to-Home Transition with a Patients Discharge Summary Tool for older adults.

Professionals represented: 2 nurses, 1 clinical nurse, 1 physiotherapist (individual interviews)

Focus group facilitator/moderator: Dr.Sc.  Joanie Pellet (investigator)

Focus group observer: Raquel Solano (research collaborator)

| Time | Section | Thèmes and Guide | Follow-up questions |
| --- | --- | --- | --- |
| 5 min | Introduction | Thank the professional for their participation.  Explain the context of the interview.  Propose and structure of the interview:  Identify individual perceptions regarding the challenges and facilitators encountered when using the PODS.  The interview will last approximately 45 minutes.  Reminder of confidentiality and consent for audio recording:  Confidentiality and use of the recording solely for transcription and analysis.  Questions? |  |
| 10 min | Open question | Experience using the PODS.  Introductory question:   - Could you tell me about your experience with the PODS since it has been implemented in the unit? | - Have you had the opportunity to use it regularly? - How did its integration into your daily practice unfold? - What were your overall impressions? - Have you encountered situations in which using it was particularly helpful? - Conversely, were there moments when you found it difficult or not very relevant to use? - Could you provide a concrete example? |
| 15 min | Specific questions | Perceived advantages and benefits   - What do you think are the main benefits that the PODS has brought to your patients/caregivers? - And for you, in your professional practice? | - Have you observed any concrete improvements in the patient ‘discharge preparation? |
|  |  | Challenges and difficulties encountered.   - What were main difficulties you encountered when using the PODS? - Were there situations in which using the PODS was more complicated? | Organizational or human barriers that may have limited its adoption.  Facilitators that helped strengthen the team’s appropriation of the PODS |
|  |  | Potential solutions/strategies   - If you could improve one thing about the use of the PODS, what would it be? - Do you have any suggestions to make its use more effective and beneficial? | What strategies have you implemented (or observed) to overcome these difficulties?  What helped facilitate its use? |
| 5 min | Closing | Introduce the end of the discussion:  We have X minutes left. Summarize the keys points and confirm their accuracy.  Ask the participant if he/she would like to add anything.  Thank them for their time and contribution. |  |

# **Supplementary material 3.** Participants flowchart

Eligible patients approached for inclusion in the control group

(n = 129)

Declined to participate (n = 63)

Included in intervention group: n = 64

Analysed (n = 56)

Analysed (n = 55)

Included in control group: n = 66

Exclusion (n = 8)

Reason for exclusion:

Lost to follow-up: n = 5

Discharge destination: n = 2

Transferred: n = 1

Exclusion (n = 11)

Reason for exclusion:

Lost to follow-up: n = 5

Discharge destination: n = 5

Hospital readmission: n = 1

Eligible patients approached for inclusion in the control group

(n = 88)

Declined to participate (n = 24)

# **Supplementary material 4.** Difficulties experienced after discharge across life domains

| Domains | Control Group (N = 23) n (%) | Intervention Group (N = 27) n (%) |
| --- | --- | --- |
| Physical health | 16 (73) | 21 (91) |
| Mobility | 11 (50) | 9 (39) |
| Mental health | 8 (36) | 10 (44) |
| Nutrition | 8 (36) | 9 (39) |
| Housekeeping | 8 (36) | 7 (30) |
| Treatment | 7 (32) | 8 (35) |
| Attendance of public places | 7 (32) | 6 (26) |
| Leisure | 7 (32) | 5 (22) |
| Administrative tasks | 5 (23) | 6 (26) |
| Finances | 4 (18) | 5 (22) |
| Personal hygiene | 3 (14) | 6 (26) |
| Work | 4 (18) | 3 (13) |
| Housing | 4 (18) | 1 (4) |
| Acquaintances and friendships | 1 (5) | 4 (17) |
| Family | 3 (14) | 2 (9) |
| Addictions | 2 (9) | 3 (13) |
| Sexuality | 4 (18) | 2 (9) |
| Other | 6 (29) | 5 (22) |

| **Supplementary material 5. Normalization Measure Development (NoMAD) questionnaire (N=30)** | | | | | | |
| --- | --- | --- | --- | --- | --- | --- |
|  | | Agree  n (%) | Disagree  n (%) | Neither agree nor disagree  n (%) | Not relevant for my role  n (%) | Not relevant at this stage  n (%) |
| 1. | I can see how the PODS intervention differs from usual ways of working | 26 (86.7) | 4 (13.3) | 0 | 0 | 0 |
| 2. | Staff in this organisation have a shared understanding of the purpose of the PODS intervention | 15 (50) | 5 (16.7) | 6 (20) | 0 | 4 (13.3) |
| 3. | I understand how the PODS intervention affects the nature of my own work | 26 (86.7) | 1 (3.3) | 2 (6.7) | 0 | 1 (3.3) |
| 4. | I can see the potential value of the PODS intervention for my work | 26 (86.7) | 3 (10) | 1 (3.3) | 0 | 0 |
| 5. | There are key people who drive the PODS intervention forward and get others involved | 16 (53.3) | 1 (3.3) | 7 (23.3) | 0 | 6 (20) |
| 6. | I believe that participating in the PODS intervention is a legitimate part of my role | 25 (83.3) | 2 (6.7) | 2 (6.7) | 0 | 1 (3.3) |
| 7. | I’m open to working with colleagues in new ways to use the PODS intervention | 24 (80) | 3 (10) | 2 (6.7) | 1 (3.3) | 0 |
| 8. | I will continue to support the PODS intervention | 23 (76.7) | 2 (6.7) | 3 (10) | 0 | 2 (6.7) |
| 9. | I can easily integrate the PODS intervention into my existing work | 21 (70) | 5 (16.7) | 2 (6.7) | 1 (3.3) | 1 (3.3) |
| 10. | The PODS intervention disrupts working relationships | 3 (10) | 18 (60) | 5 (16.7) | 0 | 4 (13.3) |
| 11. | I have confidence in other people’s ability to use the PODS intervention | 23 (76.7) | 3 (10) | 3 (10) | 0 | 1 (3.3) |
| 12. | Work is assigned to those with skills appropriate to the PODS intervention | 22 (73.3) | 3 (10) | 2 (6.7) | 0 | 3 (10) |
| 13. | Sufficient training is provided to enable staff to implement the PODS intervention | 14 (46.7) | 4 (13.3) | 7 (23.3) | 1 (3.3) | 4 (13.3) |
| 14. | Sufficient resources are available to support the PODS intervention | 11 (36.7) | 8 (26.7) | 7 (23.3) | 0 | 4 (13.3) |
| 15. | Management adequately supports the PODS intervention | 12 (40) | 2 (6.7) | 8 (26.7) | 1 (3.3) | 7 (23.3) |
| 16. | I am aware of reports about the effects of the PODS intervention | 13 (43.3) | 3 (10) | 10 (33.3) | 0 | 4 (13.3) |
| 17. | The staff agree that the PODS intervention is worthwhile | 16 (53.3) | 3 (10) | 8 (26.7) | 0 | 3 (10) |
| 18. | I value the effects that the PODS intervention has had on my work | 25 (83.3) | 2 (6.7) | 0 | 0 | 3 (10) |
| 19. | Feedback about the PODS intervention can be used to improve it in the future | 25 (83.3) | 0 | 1 (3.3) | 0 | 4 (13.3) |
| 20. | I can modify how I work with the PODS intervention | 19 (63.3) | 5 (16.7) | 4 (13.3) | 1 (3.3) | 1 (3.3) |
| 21. | Do you feel the PODS will become a normal part of your work? (0 (not at all) – 10 (comptetely)) | |  | M=6.2 | | |

# **Supplementary material 6**: Feasibility survey conducted among the healthcare team at T1: start of implementation (*n* = 20), T2: after three months of using the discharge summary (*n* = 15), and T3: after five months (*n* = 24).

# **Supplementary material 7**: Completion of PODS sections

| PODS completed sections | N=37 |
| --- | --- |
|  | n (%) |
| Hospitalization reason | 33 (89) |
| Current health issues | 30 (81) |
| Symptoms | 26 (70) |
| What to do | 15 (41) |
| Warning signs | 20 (54) |
| Changes in life habits |  |
| Type of activity | 22 (59) |
| Instruction | 16 (43) |
| Where to find more information |  |
| Who | 13 (35) |
| Contact | 8 (22) |
| Follow-up appointment |  |
| Who | 24 (65) |
| When | 16 (43) |
| Personal notes | 9 (24) |
